# Supplementary material for: Properties of Graphene-Related Materials Controlling the Thermal Conductivity of Their Polymer Nanocomposites
Source: Nanomaterials (Basel). 2020 Oct 30;10(11):2167. doi: 10.3390/nano10112167 (PMC7692405; doi:10.3390/nano10112167)
Supplement: Supplementary file 1 [file nanomaterials-10-02167-s001.pdf]

nanomaterials

Supplementary Materials

Properties of Graphene-Related Materials Controlling the Thermal Conductivity of Their Polymer Nanocomposites

Samuele Colonna, Daniele Battegazzore, Matteo Eleuteri, Rossella Arrigo and Alberto Fina *

Dipartimento di Scienza Applicata e Tecnologia, Politecnico di Torino, Alessandria Campus, Viale Teresa Michel 5, 15121 Alessandria, Italy; samuele.colonna@polito.it (S.C.); daniele.battegazzore@polito.it (D.B.); matteo.eleuteri@polito.it (M.E.); rossella.arrigo@polito.it (R.A.)

* Correspondence: alberto.fina@polito.it

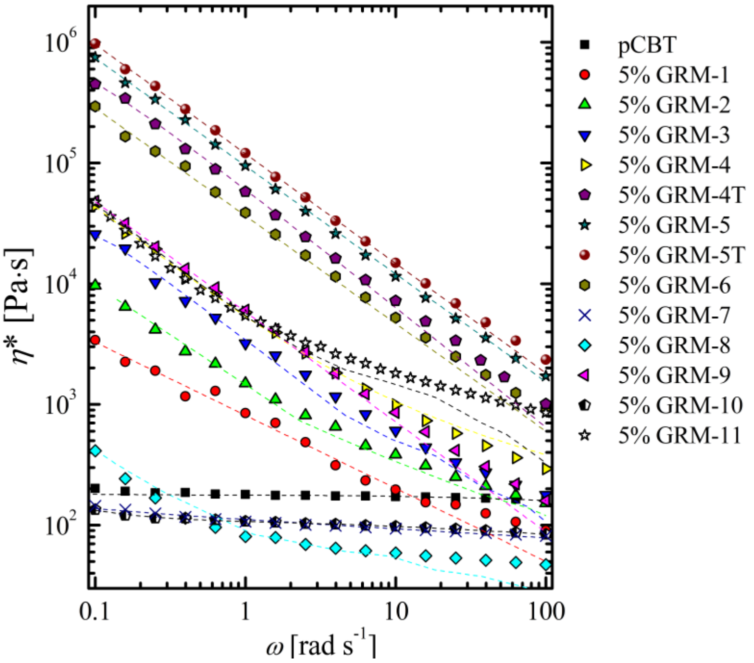

Figure S1. Fitting of experimental complex viscosity data with modified Carreau model.

© 2020 by the authors. Submitted for possible open access publication under the terms and conditions of the Creative Commons Attribution (CC BY) license (http://creativecommons.org/licenses/by/4.0/).
